# The Topoisomerase 1 Inhibitor Austrobailignan-1 Isolated from *Koelreuteria henryi* Induces a G2/M-Phase Arrest and Cell Death Independently of p53 in Non-Small Cell Lung Cancer Cells

**DOI:** 10.1371/journal.pone.0132052

**Published:** 2015-07-06

**Authors:** Chun-Chi Wu, Keh-Feng Huang, Tsung-Ying Yang, Ya-Ling Li, Chi-Luan Wen, Shih-Lan Hsu, Tzu-Hsiu Chen

**Affiliations:** 1 Institute of Medicine, Chung Shan Medical University, Taichung, Taiwan, ROC; 2 Department of Medical Research, Chung-Shan Medical University Hospital, Taichung, Taiwan, ROC; 3 Department of Applied Chemistry, Providence University, Taichung, Taiwan, ROC; 4 Division of Chest Medicine, Department of Internal Medicine, Taichung Veterans General Hospital, Taichung, Taiwan, ROC; 5 Department of Medicine, School of Medicine, National Yang-Ming University, Taipei, Taiwan, ROC; 6 Department of Medical Research, Taichung Veterans General Hospital, Taichung, Taiwan, ROC; 7 Taiwan Seed Improvement and Propagation Station, Council of Agriculture, Propagation Technology Section, Taichung, Taiwan, ROC; 8 Department of Health and Nutrition, Chia Nan University of Pharmacy & Science, Tainan, Taiwan, ROC; University of Hawaii Cancer Center, UNITED STATES

## Abstract

*Koelreuteria henryi* Dummer, an endemic plant of Taiwan, has been used as a folk medicine for the treatment of hepatitis, enteritis, cough, pharyngitis, allergy, hypertension, hyperlipidemia, and cancer. Austrobailignan-1, a natural lignan derivative isolated from *Koelreuteria henryi* Dummer, has anti-oxidative and anti-cancer properties. However, the effects of austrobailignan-1 on human cancer cells have not been studied yet. Here, we showed that austrobailignan-1 inhibited cell growth of human non-small cell lung cancer A549 and H1299 cell lines in both dose- and time-dependent manners, the IC_50_ value (48 h) of austrobailignan-1 were 41 and 22 nM, respectively. Data from flow cytometric analysis indicated that treatment with austrobailignan-1 for 24 h retarded the cell cycle at the G2/M phase. The molecular event of austrobailignan-1-mediated G2/M phase arrest was associated with the increase of p21^Waf1/Cip1^ and p27^Kip1^ expression, and decrease of Cdc25C expression. Moreover, treatment with 100 nM austrobailignan-1 for 48 h resulted in a pronounced release of cytochrome c followed by the activation of caspase-2, -3, and -9, and consequently induced apoptosis. These events were accompanied by the increase of PUMA and Bax, and the decrease of Mcl-1 and Bcl-2. Furthermore, our study also showed that austrobailignan-1 was a topoisomerase 1 inhibitor, as evidenced by a relaxation assay and induction of a DNA damage response signaling pathway, including ATM, and Chk1, Chk2, γH2AX phosphorylated activation. Overall, our results suggest that austrobailignan-1 is a novel DNA damaging agent and displays a topoisomerase I inhibitory activity, causes DNA strand breaks, and consequently induces DNA damage response signaling for cell cycle G2/M arrest and apoptosis in a p53 independent manner.

## Introduction

Lung cancer is the leading cause of death for both men and women in many countries, including Taiwan, which exhibited the highest rate of increase in lung cancer mortality in a recent decade [1, 2]. The five-year survival rate of lung cancer patients beyond stage II is only 13–25% [3]. Lung cancers are histologically classified into two major types: small cell lung cancer (SCLC) and non-small cell lung cancer (NSCLC). The NSCLC, account for 85% of the lung cancer incidence, and can be further subdivided into three groups: adenocarcinoma, squamous cell carcinoma and large cell carcinoma. Clinical strategies for treatment of lung cancer patients include surgery, chemotherapy, radiation therapy and targeted therapy. Although, promising therapy has emerged for treatment of lung cancer patients in the past decade, a large portion of patients remain uncured [4]. Therefore, to search for new drugs with greater efficacy and safety is urgently needed for lung cancer patients.

Apoptosis is a tightly regulated process controlled by either extrinsic (Death receptor) and/or intrinsic (mitochondrial) pathways [5]. The Bcl-2 family proteins have a central role in controlling the mitochondrial apoptotic pathway. Bcl-2 and Mcl-1 are anti-apoptotic members of Bcl-2 family and their elevated expression is found in many types of tumor cells [6]. Bax and Bak belong to pro-apoptotic members of the Bcl-2 family, their activation leads to oligomerization causing the formation of pores which in turn results in an increase of mitochondrial outer membrane permeability and releasing cytochrome c to activate caspase cascade. Bcl-2 and Mcl-1 can directly bind with Bax and prevent apoptotic activation of Bax [7]. PUMA is a general sensor of apoptotic stimuli and a promising drug target for cancer therapy [8, 9], which induces apoptosis by activating the pro-apoptotic protein Bax through its interaction with anti-apoptotic Bcl-2 family members, including Bcl-2 and Mcl-1. The interactions of PUMA with anti-apoptotic proteins cause displacement of Bax, resulting in activation of the pro-apoptotic activity of Bax [10]. Accumulating evidence points out that induction of apoptosis by targeting Bcl-2 family proteins is considered a potentially promising therapeutic approach in human cancers [7].

Accumulating evidence indicates that herbal medicines have anti-cancer properties and show the ability to inhibit the growth or induce the apoptosis of various types of tumor cells. The active components of herbal medicines that are responsible for the anti-cancer effects and their underlying mechanisms, however, remain largely unclear. The identification and characterization of these components, thus, might help to accelerate the development of potential anti-cancer drugs. *Koelreuteria henryi* Dummer (*K*. *henryi*), an endemic plant in Taiwan, has been used as a folk medicine for the treatment of enteritis, hepatitis, allergy, hypertension, pharyngitis, cough, hyperlipidemia, and cancer in Taiwan [11–13]. Lignan, a phytoestrogen derivative compound which widely exists in herbs, exhibits various physiological effects including the improvement of liver and cardiovascular function and the prevention of osteoporosis and cancers [14]. Lignans have also been found to be potent inhibitors of human DNA topoisomerase-I and II [15–17]. Austrobailignan-1, a natural lignin, isolated from the leaf of *K*. *henryi*, has anti-proliferative effects in various types of tumorous cells [13, 18, 19]; the effects and underlying mechanisms of austrobailignan-1 in cancer cells, however, remain unknown. In this study, we isolated austrobailignan-1 from the leaf of the *K*. *henryi*, and examined the DNA topoisomerase I inhibitory effect in vitro and cytotoxic effects of austrobailignan-1 in human non-small cell lung cancer cells. We found that austrobailignan-1 inhibited the topoisomerase 1 activity and caused DNA damage response signaling, consequently retarded the cell cycle at the G2/M phase and triggered apoptotic cell death in both lung adenocarcinoma A549 and H1299 cell lines. Besides, we also showed that multiple molecules related to cell cycle arrest and apoptosis were modulated by austrobailignan-1.

## Materials and Methods

### Chemicals and reagents

The 4’, 6’-diamindino-2-phenylindole (DAPI), Propidium iodide (PI), ribonuclease (RNase), dimethyl sulfoxide (DMSO), and Triton X-100 were purchased from Sigma-Aldrich Inc. (St. Louis, MO. USA). Fetal Bovine serum and RPMI1640 medium were purchased from GIBCO BRL (Gaithersburg, MD, USA). The antibodies against p21^Waf1/Cip1^ (sc-397), p27^Kip1^ (sc-667), p53 (sc-126), Cdc25c (sc-327), Cdk1 (sc-53219), cyclin A1 (sc-751), cyclin B1 (sc-245), Bcl-2 (sc-509), cytochrome c (sc-13156) and β-actin (sc-47778) were purchased from Santa Cruz Biotechnology (Santa Cruz, CA, USA). COX IV (4844), Mcl-1 (4572), Bax (2772), Bak (3814), PUMA (12450), phospho-ATM (4526), phospho-H2AX (9718), phospho-Chk1 (2341), phospho-Chk2 (2661) and phospho-p53 (9284) antibody were purchased from Cell Signaling Technology (Danvers, MA, USA) as described in previous report [20]. The inhibitor of caspase-2 (Z-VDDADFMK, #FMK003) was purchased from R&D system (MN, USA). The inhibitor of caspase-3 (Z-DEVD-FMK, #550378) or caspase -9 (Z-LEHD-FMK, # 550381) was purchased from BD Bioscience (MD, USA).

### Cell lines and cell culture

The human non-small cell lung cancer cell lines, including A549, H1299, A549-shRNA and A549-p53shRNA provided by Dr. Hsu-Shih-Lan [21] were maintained in RPMI 1640 medium with 5% heat-inactivated fetal bovine serum, and incubated in a 5% CO_2_ incubator at 37°C.

### Plant material

The leaves of *K*. *henryi* were collected from Taiwan by Dr. Chi-Luan Wen, Taiwan Seed Improvement and Propagation Station, Council of Agriculture, Propagation Technology Section, where a voucher specimen was deposited.

### Isolation and purification of austrobailignan-1

**Fig 1:**
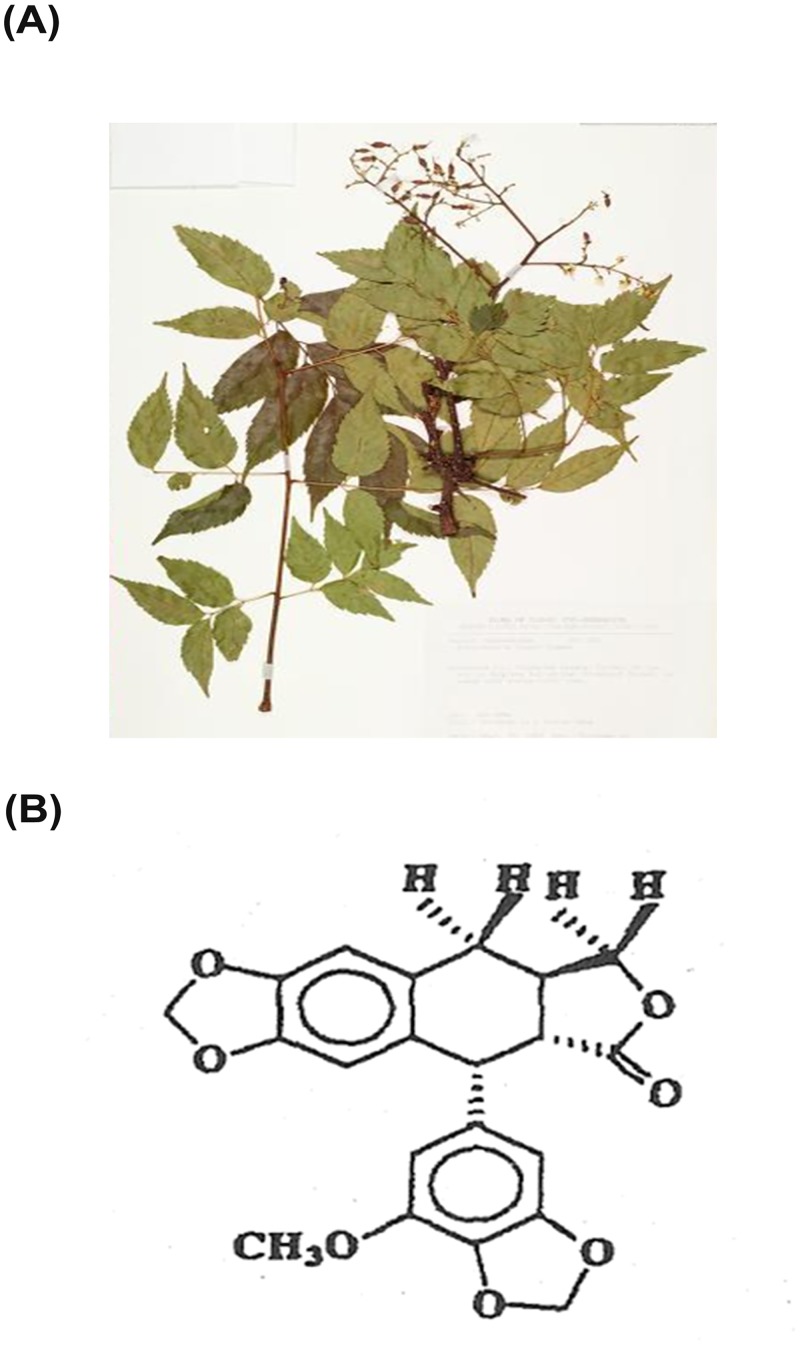
The structure pf austrobailignan-1 from the leaves of *K*. *henryi* Dummer. (A) The image of K. henryi Dummer (adopted from Herbanum, Academic Sinica, http://digiarch.sinica.edu.tw/content/repository/resource_content.jsp?oid=3819630). (B) The structure of austrobailignan-1 was established via ^1^H- and ^13^C-NMR. ^13^C-NMR assignments were based on HETCOR and long-range HECTOR spectral results.

The austrobailignan-1 used in this study was extracted and purified from the leaves of *K*. *henryi* (Fig 1A) according to the processes described elsewhere with minor modification [12]. Briefly, the dried leaves (1 kg) of *K*. *henryi* were milled and extracted by 95% ethanol three times at room temperature. The ethanol extract was partitioned with H_2_O-CH_2_Cl_2_ (1:1) mixture, and then the CH_2_Cl_2_ fraction was collected and dissolved in 90% methanol followed by extraction with hexane. The methanol fraction was harvested and chromatographed on a silica gel column, using hexane, hexane/CHCl_3_ and CHCl_3_/methanol as the eluting solvent followed by thin layer chromatography to collect the cytotoxic fractions. These fractions were pooled and then run through a silica gel column, eluted with a gradient of hexane/EtOAc and followed by a reversed-phase C8 Labor column to yield austrobailignan-1 followed by lyophilization as powder with the purity higher than 90% [22]. The powder was then dissolved in DMSO at a stock concentration of 100 μM for experimental applications. The structure of austrobailignan-1 (Fig 1B) was identified by 2D ^1^H- and ^13^C-NMR based on HETCOR and long-range HETCOR spectral data, and direct comparison with the previous data of related lignans [23].

### Cell proliferation and cell cycle distribution analysis

A549, A549-shRNA, A549-p53shRNA and H1299 lung cancer cells were seeded in 12-well plates (2 x 10^4^ cells/well). After 24 h, cells were treated with austrobailignan-1 for the indicated time period followed by a trypan blue exclusion assay. In addition, to analyze the cell cycle distribution, the austrobailignan-1-treated cells were fixed with ethanol, and then stained with propidium iodide followed by subjecting to a flow cytometric assay to determine the cell cycle distribution ratio.

### Terminal deoxynucleotide transferase dUTP nicked-end labeling (TUNEL) assay

The A549 or H1299 cells were treated or untreated with austrabalignan for 24 h. The cells were then subjected to TUNEL assay as described else in previous report [24].

### Caspase activity assay

The austrobailignan-1-treated or untreated A549 cells were collected, lyzed and subjected to the caspase activity assay as described else in previous reports [24, 25].

### Immunoblotting

The extraction of cell lysates were described in previous report (ref), Briefly, the cells were extracted with RIPA lysis buffer (150 mM NaCl, 10 mM Tris, pH 7.5, 1% NP-40, 1% deoxycholate, 0.1% SDS, and protease inhibitor cocktail). The lysates were then separated by the SDS-PAGE, and then transferred to the PVDF membrane followed by the western blot procedure using indicated antibodies. Image was resolved by chemiluminescence.

### Protein subcellular fractionation

The experiment was described else in previous report [26]. Briefly, the parental or austrobalignan-1-treated cells were lyzed with sucrose buffer (2 mM EDTA, 250 mM sucrose, 10 mM DTT and 2 mM EGTA) on ice for 30 min. Lysates were then centrifuged at 1300*g* for 10 min at 4°C. The supernatant was further centrifuged 100,000 xg for 30 min at 4°C to collect the cytosolic fraction. The heavy membrane pellet was then resuspended in 1 mL of sucrose buffer and laid on the top of 1.2 and 1.5 M sucrose gradient buffer followed by centrifuging at 1300*g* for 30 min at 4°C. The enriched mitochondria fractions were collected at 1.2/1.5 M interphases respectively, followed by dissolving in RIPA lysis buffer.

### Comet assay

The A549 and H1299 cells were treated without or with 30 and 100 nM austrobailignan-1 for 24 h and the comet assay procedure including slide preparation, lysis, alkaline incubation, electrophoresis, neutralization and visualization was carried out as described elsewhere [27]. Ethidium bromide labeled DNA was visualized under a fluorescence microscope. The degree of DNA damage was scored by tail moment (% DNA in tail x tail length) from at least 100 cells in each treatment group.

### *In vitro* DNA topoisomerase 1 inhibition assay

DNA topoisomerase 1 activity was determined by measuring the relaxation of supercoiled plasmid DNA as described elsewhere with minor modification [28]. Briefly, various concentrations of austrobailignan-1 were added to the topoisomerase-1 activity reaction mixture (including 250 ng pHOT-1 plasmid DNA, 0.3 U calf thymus DNA topoisomerase 1, 20 mM Tris-HCl, pH 8.0, 72 mM KCl, 5 mM MgCl_2_, 5 mM dithiothreitol, 2 mM spermidine, 0.01% bovine serum albumin) for 30 min at 37°C, and the reaction was stopped by loading dye solution (2.5% SDS, 25 mM EDTA, pH 8.0, 15% ficoll-400, 0.05% bromophenol blue, and 0.05% xylene cyanole). The reaction products were separated by 1% agarose gel electrophoresis, and visualized by staining with ethidium bromide. CPT (camptothecin) was used as a positive control.

### Statistical Analyses

All data were showed as mean±S.D. Each result was obtained at least three separate experiments. Statistical comparisons were evaluated by means of one-way analysis of variance (ANOVA), and significance was determined using student’s *t*-test and presented as *p<0.05, **p<0.01, ***p<0.001.

## Results

### Austrobailignan-1 induced cell cycle G2/M phase arrest and cell death in both A549 and H1299 cells

**Fig 2:**
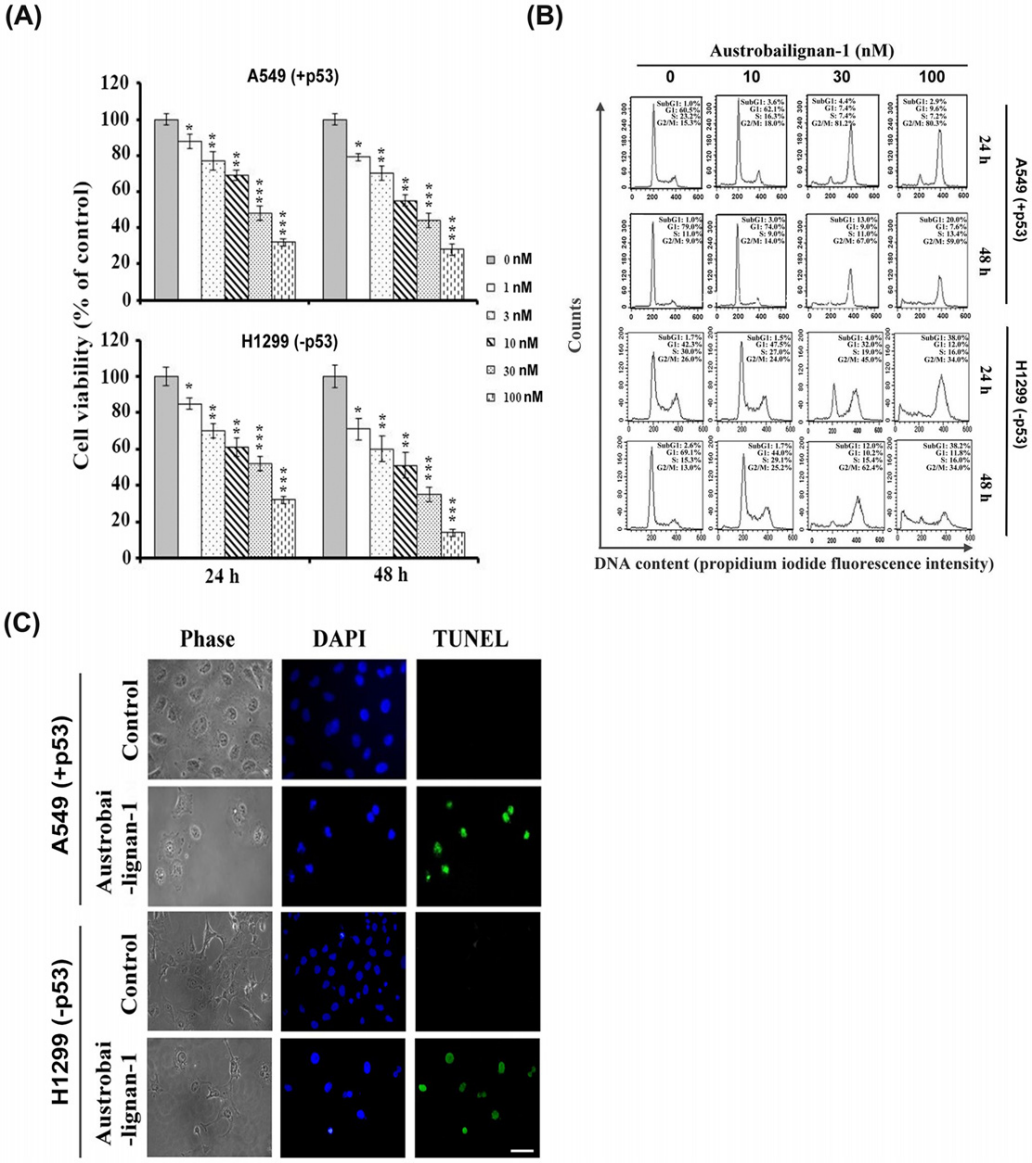
Austrobailignan-1 induced G2/M arrest and apoptosis. (A) A549 and H1299 cells were treated with various doses (0, 1, 3, 10, 30 and 100 nM) of austrobailignan-1 for 24 and 48 h. Cell number was measured by a Trypan-blue dye exclusion method. Data are expressed as mean ± S.D. from 3 independent experiments. (*P <0.05,** P <0.01, *** P < 0.001 v.s. control). (B) Cells were treated with varied doses (0, 3, 10, 30 and 100 nM) of austrobailignan-1 for 24 and 48 h, and then stained with propidium iodide, and flow cytometry was performed to examine the cell cycle distribution. (C) Cells were treated without or with 100 nM austrobailignan-1 for 48 h,a TUNEL assay was then performed to detect apoptotic cells (green) and the nuclear DNA was stained with DAPI (blue). The stained cells were investigated by fluorescence microscopy. Magnification x 400; scale bar, 50 μm.

The loss of normal function of p53 had been finding in over half of all human tumors [29]. Literature shows that p53 is one of the most important regulators in mediating growth arrest and apoptosis induced by various intrinsic or extrinsic stresses, including chemotherapeutic agents [30]. Besides, the p53 is also an important connector and switcher between cell cycle arrest and apoptotic process. Once the damages are unable to be repaired, p53 activates the transcription of various pro-apoptotic genes, including Bax, Noxa, PUMA, Fas, and DR5 [31, 32] to execute the apoptotic process. Alternatively, p53 triggers apoptosis by repression of anti-apoptotic genes, such as Bcl-2, thus inducing the release of cytochrome c followed by the caspase-3 and -9 activation [31]. It is well documented that the status of p53 can affect the response of cancer cells to some chemotherapeutic drugs [33]. We firstly examined the antiproliferative effects of austrobailignan-1 purified from the leaves of *K*. *henryi* (Fig 1 and [12]) in human NSCLC A549 (+p53, which harvest a wild-type p53) and H1299 (-p53, which is p53-null) cell lines. As shown in Fig 2A, treatment with austrobailignan-1 significantly inhibited the growth of A549 and H1299 cells in both concentration- and time-dependent manners with IC_50_ values of 41 and 22 nM after 48-h administration, respectively. The results also showed that treatment of lung cancer cells with low concentrations (lower than 10 nM) of austrobailignan-1 caused a cytostatic effect, only inhibited cell proliferation but no cytotoxic effect observed under microscopic investigation. However, treatment with high concentration (30 and 100 nM) of austrobailignan-1 exhibited morphological features of apoptotic cell death, floating and blebbing cells were observed (data not shown). To address the precise action responsible for the austrobailignan-1-mediated antiproliferative effect, the cell cycle distribution profile was examined. As indicated in Fig 2B, exposure of A549 and H1299 cells to 30 and 100 nM of austrobailignan-1 for 24 h led to an accumulation of cells in the G2/M phase compared with control cells, coupled with a concomitant decrease in the proportion of cells in the G1 and S phases. Additionally, a hypodiploid DNA content peak (sub-G1 population), which is indicative of degraded DNA and a hallmark of apoptosis, was observed following 24 h of high-dose treatment and increased continuously after 48-h austrobailignan-1 incubation (Fig 2B). To further confirm the induction of apoptosis by austrobailignan-1 in A549 cells, the TUNEL assay and DAPI staining were performed. As indicated in Fig 2C, treatment with 100 nM austrobailignan-1 for 48 h significantly induced the apoptotic cell death with condensed nuclei and increase of TUNEL positive cells (green fluorescent colored cells), suggesting that the DNA fragmentation was occurring in these cells.

### Austrobailignan-1 inhibited topoisomerase 1 activity and induced the DNA damage signaling pathway

**Fig 3:**
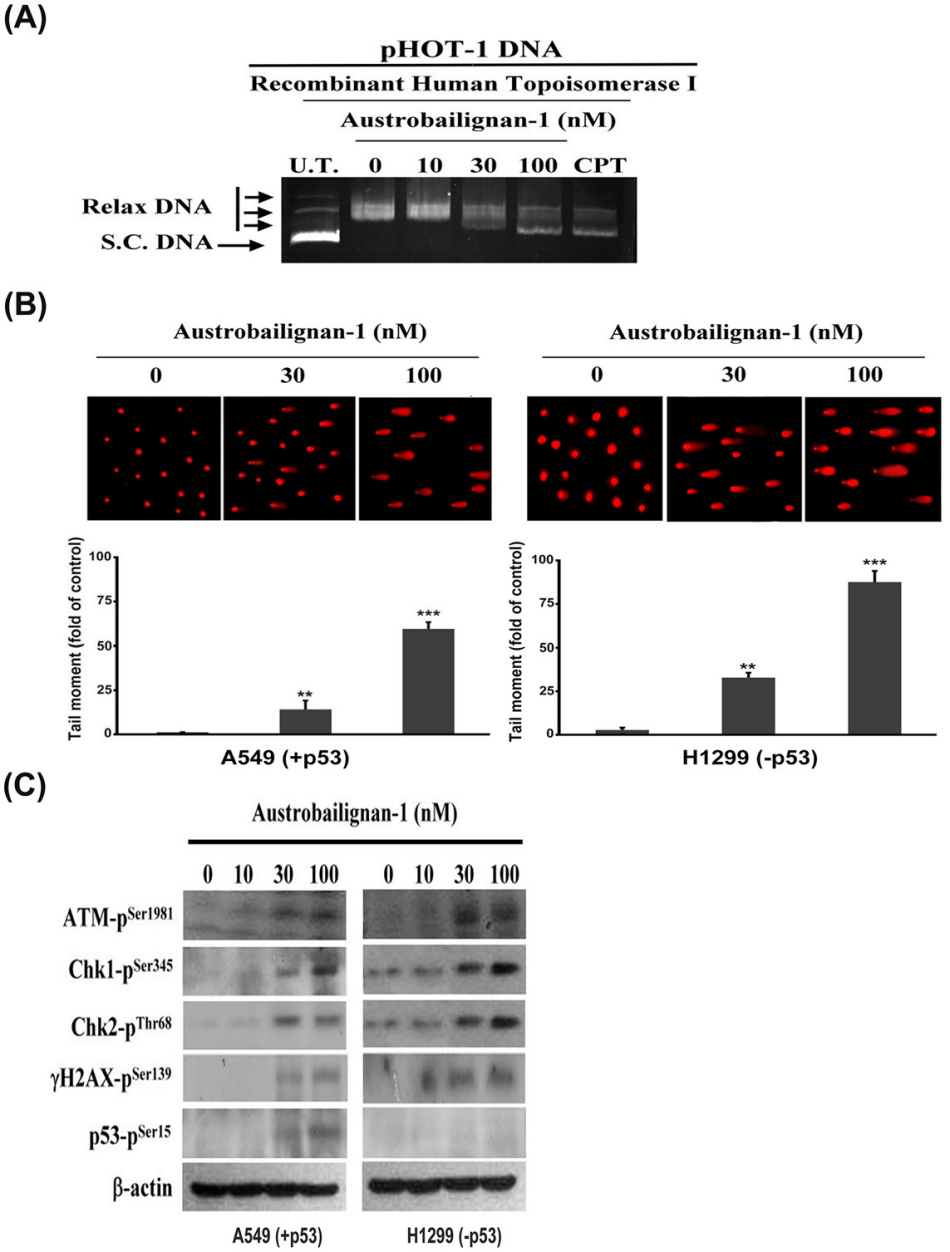
Austrobailignan-1 inhibited topoisomerase 1 activity and induced a DNA damage signaling pathway. (A) Inhibition of DNA topoisomerase 1 activity. pHOT-1 DNA plasmid was incubated with various concentrations of austrobailignan-1 (0, 10, 30, and 100 nM) and topoisomerase 1 at 37°C for 30 min. The reaction products were separated by 1% agarose gel and stained by ethidium bromide. The fluorescence image was recorded by microphotography. Camptothecin (CPT) was used as a positive control. S. C. DNA: super coiled DNA, Relax DNA: unwind closed circular DNA. (B) DNA damage response. A549 and H1299 cells were treated without or with 30, 100 nM austrobailignan-1 for 24 h, and DNA damage on per cell basis was examined by a comet assay. Representative comet images from the cells exposed to austrobailignan-1 at various concentrations are shown (upper panel). The degree of DNA damage was scored by tail moment (% DNA in tail x tail length) from at least 100 cells in each treatment group (lower panel). Data are mean ± SD for three independent experiments. ** *p* < 0.01, *** *p* < 0.001. (C) Activation of ATM signaling pathway by austrobailignan-1. A549 and H1299 cells were treated with various concentrations of austrobailignan-1 for 24 h, the expressed levels of phosphorylated ATM, Chk1, Chk2, H2AX, and p53 proteins were investigated by Western blot analysis. β-actin was used as an internal loading control.

Lignan family compounds have been found to be potent inhibitors of human DNA topoisomerase 1 [16, 17]. Next, we used a commercial DNA relaxation assay kit for in vitro measurement of topoisomerase 1 activity in the presence of austrobailignan-1. This kit is majorly to analyze the ability of topoisomerase-1 to unwind a supercoiled DNA. Fig 3A shows that austrobailignan-1 inhibited the DNA relaxation activity of topoisomerase 1 dose-dependently. Camptothecin, a known Topoisomerase 1 inhibitor, was used as the positive control. At 100 nM, austrobailignan-1 exhibited equipotent inhibitory activity to camptothecin (100 μM), indicating that austrobailignan-1 might be more effective than camptothecin.

Literature shows that topoisomerase 1 inhibitor can induce double-strand breaks (DSBs) and then lead to DNA damage response [34, 35]; therefore, a comet assay was performed to examine whether austrobailignan-1 caused DNA damage in A549 and H1299 cells. As depicted in Fig 3B, austrobailignan-1 increased the comet tail movement in both tested cells in a concentration-dependent manner. ATM is a well-known DNA damage sensor and regulator. Following exposure to DNA damage stresses such as oxidative stress or inhibitors of topoisomerase 1 and 2, ATM/ATR kinases are activated by phosphorylated at ser1981 [36], which in turn phosphorylates numerous downstream substrates, including Chk1-ser345, Chk2-thr68, γH2AX-ser139, and p53-ser15, etc., and ultimately leading to the cell cycle arrest and apoptosis [37, 38]. Next, the potential effects of austrobailignan-1 on the ATM signaling pathway were examined. Data from Western blot analysis clearly showed a concentration-dependent phosphorylation of ATM-ser1981, Chk1-ser345, Chk2-thr68, γH2AX-ser139 and p53-ser15 in austrobailignan-1-treated cells (Fig 3C). However, the levels of total ATM, Chk1, and Chk2 remained unchanged in response to austrobailignan-1 exposure (data not shown).

### Austrobailignan-1 regulated cell cycle related proteins

**Fig 4:**
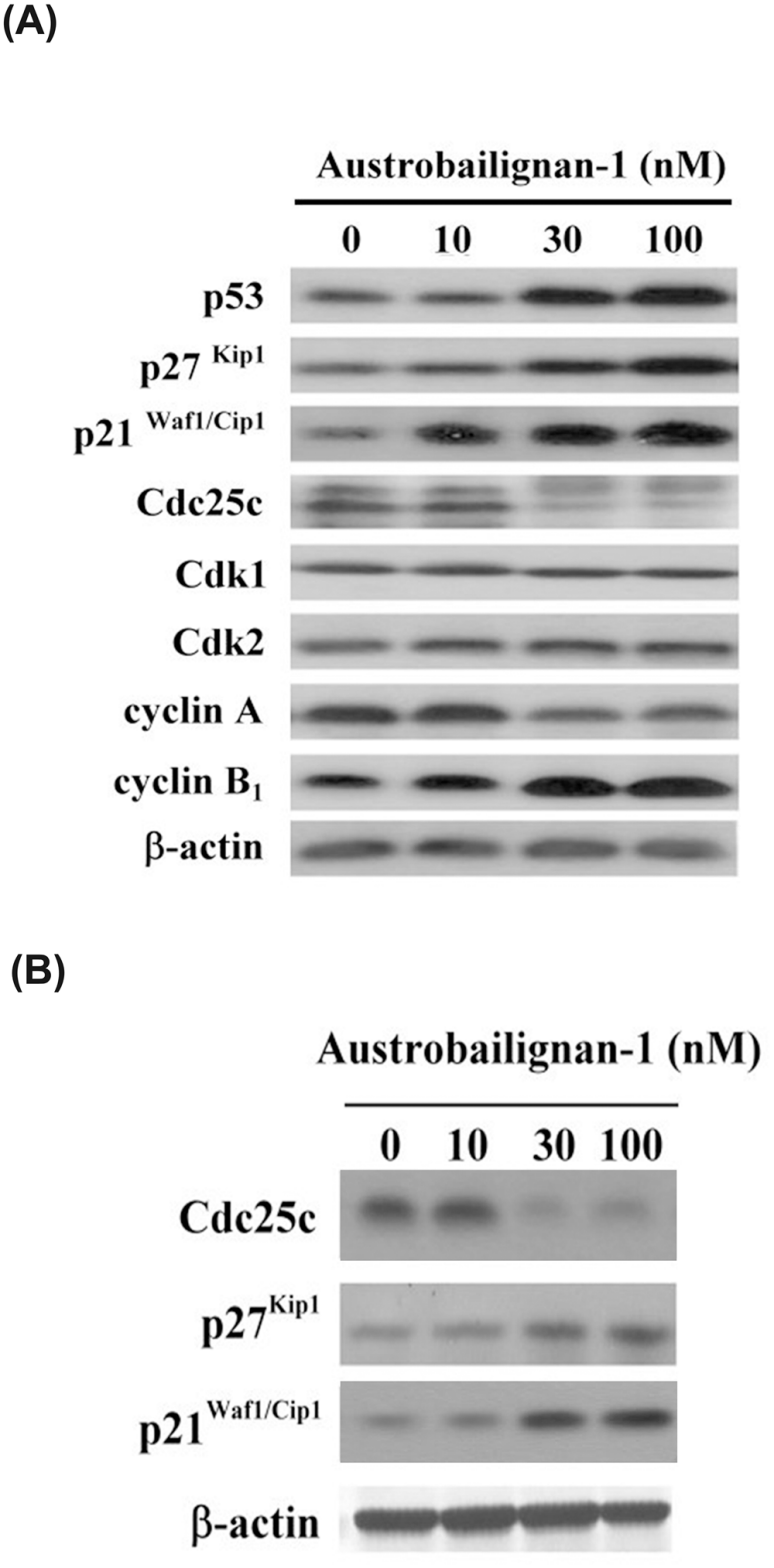
Regulation of cell-cycle regulatory proteins by austrobailignan-1. (A) A549 cells were treated with 0, 3, 10, 30 and 100 nM of austrobailignan-1 for 24. After treatment, cell extract was collected and analyzed by Western blot. (B) H1299 cells were treated with 0, 10, 30, 100 nM austrobailignan-1 for 24 h, the levels of p21Waf1/Cip1, p27Kip1, and Cdc25C were detected by Western blot. β-Actin was used as a loading control.

We have showed that p53 can be phosphorylated by ATM/ATR kinases in the presence of austrabailignan-1 in A549 cells. The active p53 can transcriptionally increase the expression levels of p21^Waf1/Cip1^, p27^Kip 1^ [39], which both are breakers of cell cycle progression. Besides, the Cdc25 dual specificity phosphatase family (Cdc25A, Cdc25B and Cdc25C) is another common signal transducer downstream substrate of ATR/ATR/Chks. Phosphorylated inactivation of Cdc25C mediated by ATM/ATR/Chks plays a pivotal role in G2/M phase arrest and subsequently apoptosis induced by several antitumor agents [40–43]. To address the subsequent molecular event of the austrobailignan-1-mediated cell cycle retardation, the expression levels of G2/M-related molecules such as p53, p27^Kip 1^, p21^waf1/Cip1^, Cdk1, Cdk2, cyclin A, cyclin B1 and Cdc25C were examined after various doses of austrobailignan-1 (0, 10, 30, and 100 nM) treatment of A549 cells for 24 h. As expected, the expressions of p53, p21^Waf1/Cip1^, p27^Kip1^ and cyclin B1 were increased while cyclin A and Cdc25C were decreased (Fig 4A) in austrobailignan-1-treated cells compared to untreated control cells. The levels of Cdk1 and Cdk2 were not affected by austrobailignan-1. Limited by the compound availability, only p21^Waf1/Cip1^, p27^Kip 1^ and Cdc25C levels were examined in p53-null H1299 cell line. Similarly, the up-regulation of p21^Waf1/Cip1^ and p27^KIP 1^ and down-regulation of Cdc25C were observed in austrobailignan-1-treated H1299 cells (Fig 4B). These results indicated that austrobailignan-1-mediated cellular and molecular events in the tested cell lines were p53 independently.

### Austrobailignan-1 induced intrinsic mitochondria-mediated apoptosis

**Fig 5:**
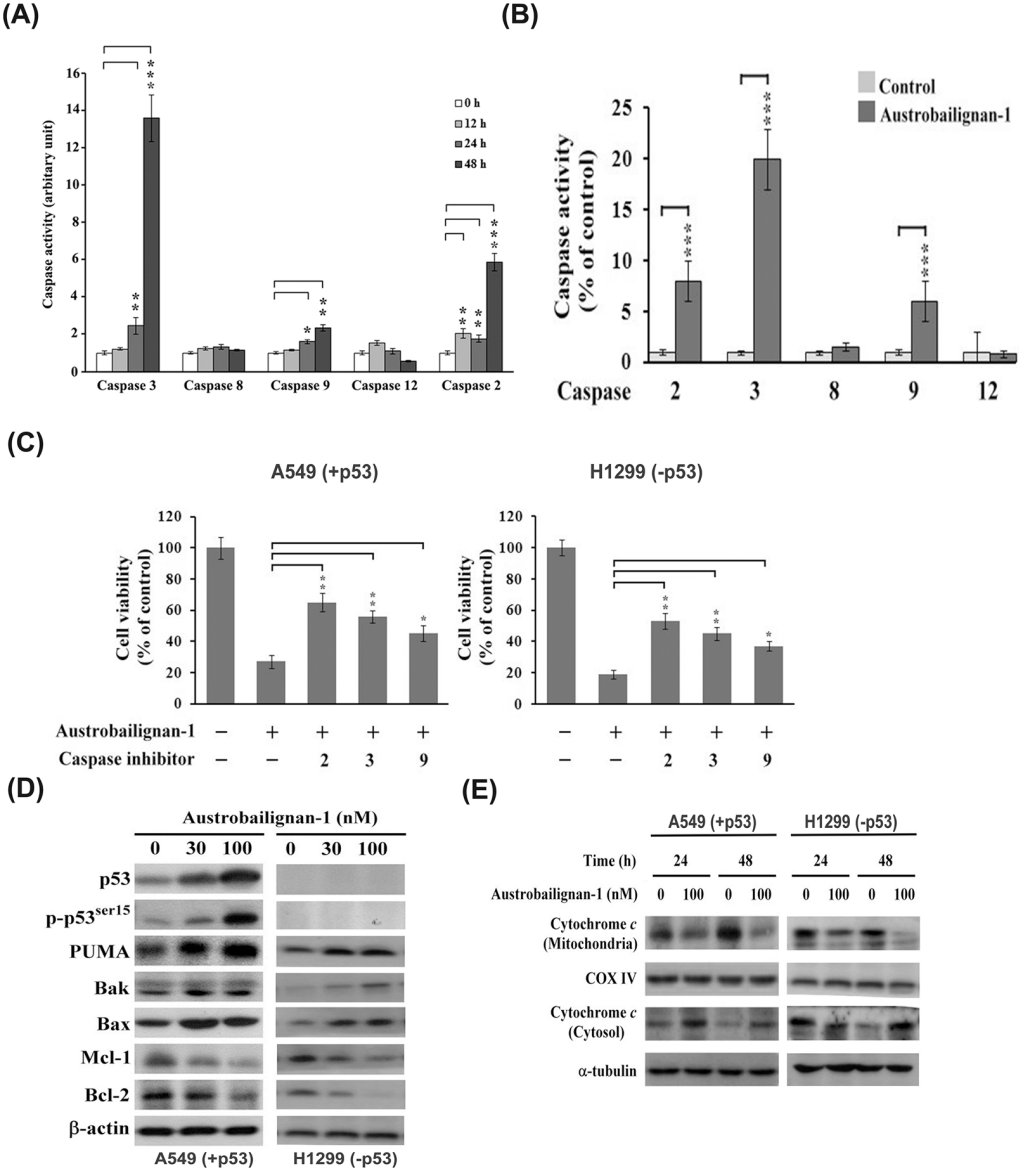
Induction of mitochondrial apoptotic pathway by austrobailignan-1. (A) A549 cells were treated with 100 nM of austrobailignan-1 for indicated time periods (0, 12, 24, and 48 h). (B) H1299 cells were exposed to austrobailignan-1 for 48 h. The cell lysates were harvested, and the caspase activities were determined using fluorogen substrates. Data are expressed as mean ± S.D. from three independent experiments. (*P <0.05,** P <0.01, *** P < 0.001 v.s. vehicle-treated control). (C) Caspase inhibitors block austrobailignan-1-induced cell death. A549 and H1299 cells were pretreated with 50 μM indicated caspase inhibitors for 1 h, and then treated with 100 nM austrobailignan-1 for another 48 h. The cell viability was measured by a Trypan blue dye exclusion method. (D) Regulation of Bcl-2 family proteins. A549 and H1299 cells were treated with 0, 30, 100 nM austrobailignan-1 for 48 h. The levels of indicated Bcl-2 family proteins were examined by Western blot. (E) Release of cytochrome *c* from mitochondria to cytosol. A549 and H1299 cells were treated without or with 100 nM austrobailignan-1 for 24 and 48 h. After treatment, particulate and cytosolic fractions were isolated, the level of cytochrome *c* protein was analyzed by Western blot. α-tubulin and cytochrome oxidase IV were used as loading control.

Because caspase activation plays a central role during the executional phase of apoptosis [44], to examine whether austrobailignan-1 induced apoptosis through activation of the caspase pathway, the activity of caspases-2, -3, -8, -9, and -12 was estimated using a caspase fluorogenic peptide substrate kit. As shown in Fig 5A and 5B, treatment of A549 and H1299 cells with 100 nM austrobailignan-1 resulted in the activation of mitochondria-related caspase-2, -9, and -3, but not death receptor-related caspase-8 and endoplasmic reticulum-associated caspase-12. To characterize the role of caspase activation in austrobailignan-1-induced apoptosis, A549 and H1299 cells were pretreated with inhibitors of caspase-2 (Z-VDDADFMK), caspase-3 (Z-DEVD-FMK), and caspase-9 (Z-LEHD-FMK) for 1 h, and then treated with 100 nM austrobailignan-1 for another 48 h. The inhibitors of caspase-2, -3, and -9 significantly protected A549 and H1299 cells against austrobailignan-1-mediated cell death (Fig 5C). These results suggested that the activated caspases might contribute to austrobailignan-1-triggered apoptosis in these cells.

On the basis of the above results, activation of caspase-2, -3, and -9 involved in austrobailignan-1-induced apoptosis, indicating that the mitochondrial apoptotic pathway was activated. It is known that Bcl-2 family proteins are involved in intrinsic mitochondria-mediated apoptosis [45]. To gain further insights into the molecular events involved in austrobailignan-1-induced apoptosis, the expressed levels of Bcl-2, Mcl-1, Bax, Bak, and PUMA were examined. The expression of pro-apoptotic molecules Bax, Bak, and PUMA was drastically increased, while the levels of anti-apoptotic proteins Bcl-2 and Mcl-1 were decreased following austrobailignan-1 treatment (Fig 5D). Cytochrome c plays a key role in Bcl-2 family protein-mediated apoptotic death. It normally resides in the mitochondria but translocates into the cytosol, driving caspase activation at the onset of apoptotic stresses. Thus, the cellular distribution of cytochrome c was investigated. As depicted in Fig 5E, administration of austrobailignan-1 resulted in releasing mitochondrial cytochrome c to cytosol. These results suggest that austrobailignan-1-induced apoptosis was mainly via the intrinsic mitochondrial-triggered pathway.

### p53 was not necessarily required for austrobailignan-1-induced cell cycle G2/M arrest and cell death

**Fig 6:**
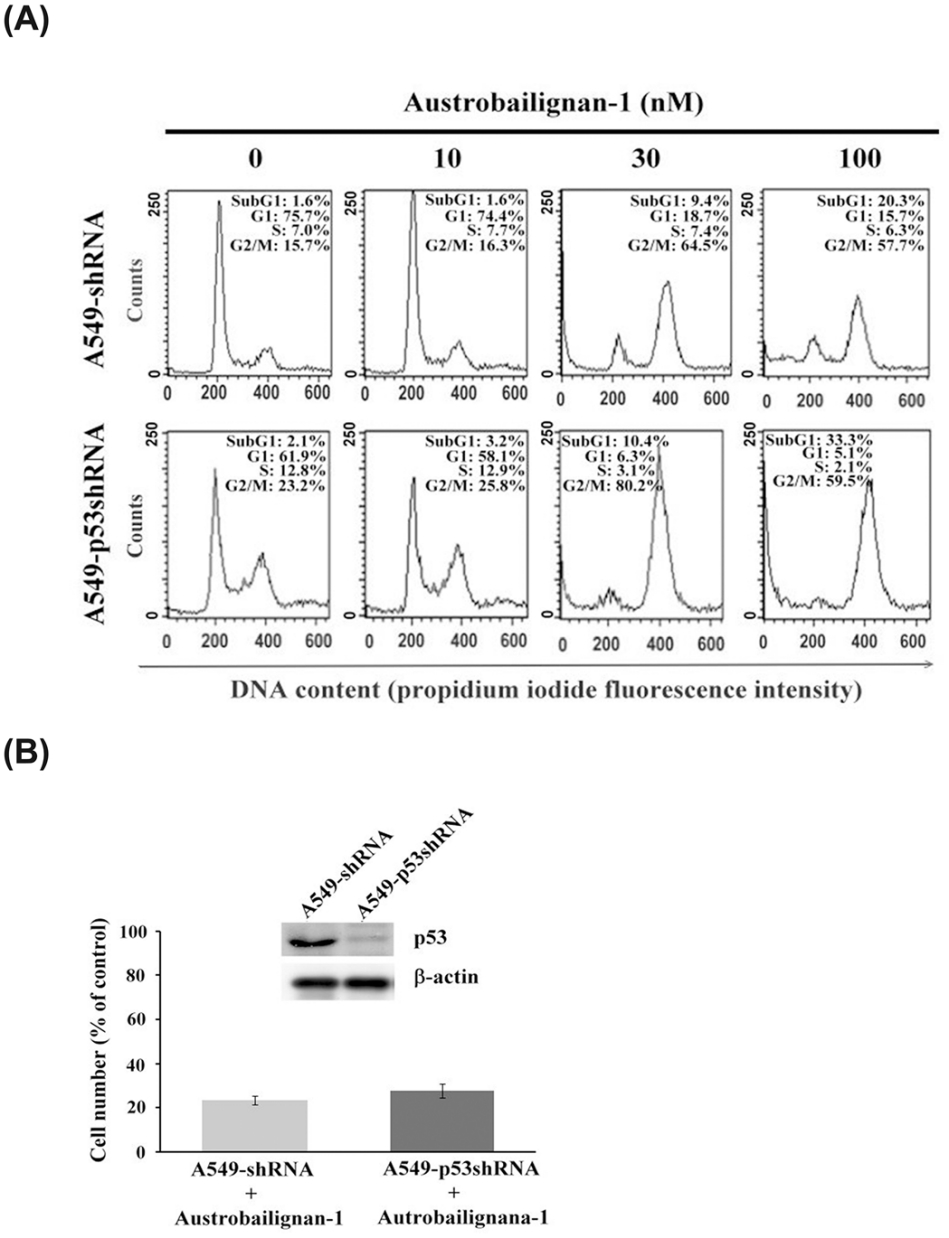
p53 was not necessarily required for austrobailignan-1-induced cell cycle G2/M arrest and cell death. (A) The A549-shRNA or A549-p53shRNA cells were treated with austrobailignan-1 (0, 10, 30, and 100 nM) for 48 h. The cell cycle distribution was determined by flow cyotmetry. (B) A549-shRNA and A549-p53shRNA cells were treated without or with 100 nM austrobailignan-1 for 48 h. The levels of p53 protein were detected by Western blot (upper panel). The cell viability was determined by a trypan blue exclusion method (lower panel).

Our results have showed that autrobailignan-1 induced cell death in both A549 and H1299 cell lines (Fig 2A and 2B). The phosphorylation of p53 at ser^15^ site usually represents apoptotic activation [46]. Figs 4A and 5E showed that austrobailignan-1 treatment increased the levels of total p53 and phosphorylated p53-ser15 in A549 cells, implying a role of p53 in austrobailigan-1-induced cell death. To characterize whether p53 indeed plays a role in austrobailignan-1-mediated cell cycle arrest and apoptosis, we next examined the effect of austrobailignan-1 in p53-knockdown A549 (A549-p53-shRNA) cells, which were stably transfected with a p53-specific shRNA [47]. There was no significant difference between A549-vector control cells (A549 (-shRNA) and A549-p53-shRNA cells in cell cycle arrest (Fig 6A) and in cell viability after 48 h austrobailignan-1 treatment (Fig 6B). We therefore conclude that austrobailignan-1-mediated G2/M arrest and cell death does not necessarily require p53.

## Discussion

In this study, we provide the evidence showing that austrobailignan-1, a lignan family compound isolated from *Koelreuteria henryi* Dummer, inhibited the topoisomerase-1 activity, and then triggered a DNA damage signaling pathway, consequently led to cell-cycle arrest and apoptosis in human non-small cell lung cancer A549 and H1299 cells. The increasing levels of p21^Waf1/Cip1^ and p27^Kip1^, and decreasing Cdc25C level, indicated that these molecules were actively involved in the response to austrobailignan-1-induced G2/M arrest. Moreover, the mitochondrial apoptosis related molecules such as Bcl-2 family proteins, cytochrome c, caspase-2, 3 and 9, contributed to austrobailignan-1-triggered apoptotic cell death.

DNA topoisomerase 1 regulates the topological state of DNA in many cellular processes, including DNA replication and transcription [48, 49]. Compounds that inhibit topoisomerase 1 activity have been widely used as anticancer drugs because blocking topoisomerase 1 activity can cause DNA damage and initiate DNA checkpoints that trigger cell cycle arrest and subsequently induce cell death [34, 50]. A well-known topoisomerase 1 inhibitor, camptothecin and its derivatives, topotecan, irinotecan and belotecan have been used clinically in a number of therapeutic protocols for many years for various types of cancer [51–53], including human non-small cell lung cancer [54, 55]. Previous studies indicate that lignans are potent inhibitors of human DNA topoisomerase 1 and 2 [16, 50]. Austrobailignan has been shown to inhibit topoisomerase activity and induce cell death in human colon carcinoma and human breast carcinoma cell lines [17]. Using an *in vitro* DNA relaxation assay, alkaline gel electrophoresis comet assay and ATM and γH2AX western blot analysis, we found that austrobailignan-1 is a potent topoisomerase 1 inhibitor. Treatment of A549 and H1299 cell lines with austrobailignan-1 exhibited similar cellular and molecular response patterns, including DNA damage, ATM, Chk1/Chk2 activation, H2AX phosphorylation (γH2AX), G2/M arrest, caspase activation, and apoptosis. Consistently, previous studies demonstrated that topoisomerase 1 inhibitors can bring about irreversible DNA damage, resulting in G2/M arrest and apoptosis, which is related to activation of ATM/γH2AX, and caspase pathways [35, 56, 57].

Cell cycle blockade is considered an effective strategy for eliminating cancer cells. It is well known that cell cycle progression is stringently regulated by the reciprocal actions between activators and inhibitors. The eukaryotic cell cycle progression is regulated by the coordinated activity of cyclin-dependent kinase (Cdk) and cyclin complexes [58]. The G2 /M transition is largely dependent on cyclin B1 / Cdk1 activity. The activity of cyclin B1/Cdk1 can be regulated by an activator, Cdc25c, and inhibitors including p53, p21^WAF1/CIP1^ and p27^KIP1^. p21^Waf1/Cip1^ and p27^Kip1^ are known Cdk inhibitors which affect G2/M cycle progression in various types of cancer cells [59, 60]. A previous study demonstrates that DNA damage signaling can increase p21^Waf1/Cip1^ expression through the p53-dependent and -independent pathways to trigger cell cycle arrest in G2 phase [33]. In this study, we showed that induction of p21^Waf1/Cip1^ and p27^Kip1^ expression was accompanied by G2/M blockade in austrobailignan-1-treated A549 and H1299 cells, suggesting that this compound-induced G2/M arrest was probably via upregulation of p21^Waf1/Cip1^ and p27^Kip1^ expression.

Previous report indicated that a novel aroylthiourea analogue-induced proliferation inhibition of human colon cancer HCT116 cells and G2/M phase arrest is involved in activation of Chk1 and inactivation of Cdc25C [41]. Jaceosidin inactivates Cdc25C-Cdk1 through ATM-Chk1/Chk2 activation, resulting in cell cycle G2/M arrest in endometrial cancer cells [61]. In this study, we found that austrobailignan-1 increased the phosphorylation of ATM, Chk1, and Chk2 and induced G2/M arrest in both A549 and H1299 cells. This event was accompanied by decreased Cdc25C protein level, which indicated that austrobailignan-1-induced G2/M arrest may also be mediated by the activation of the ATM-Chk1/Chk2-Cdc25C signaling axis. Our results are similar with the known topoisomerase I inhibitors, irinotecan and topotecan, which usually cause DNA damage and then followed by activation of ATM/Chks, decrease of Cdc25C expression, increase of p21^Waf1/Cip1^ expression, and consequently leading to G2/M arrest [57, 62].

Literature shows that activation of signaling pathways after DNA damage induced by topoisomerase inhibitors lead to trigger mitochondrial apoptotic cell death in various types of human cancer cells [63]. In the present study, austrobailignan-1-induced apoptosis of A549 and H1299 cells was confirmed by the TUNEL assay and activation of caspases (Figs 2 and 5). Treatment with austrobailignan-1 induced the activation of mitochondrial-related caspase-2, -3 and -9, but not receptor or endoplasmic reticulum-related-caspase-8 and -12, indicating that austrobailignan-1 induces apoptosis mainly via a mitochondrial- dependent manner. Despite the activation of caspase-2 mainly via p53-dependent PIDD pathway under DNA damage, it is still unable to rule out the possibility that ATM/ATR activate caspase-2 via a p53-independent Chk1 manner [64]. Furthermore, the release of mitochondrial cytochrome c, decrease of anti-apoptotic proteins (such as Bcl-2 and Mcl-1) and increase of pro-apoptotic proteins (including Bax and PUMA) may further support the theory that austrobailignan-1-induced apoptosis is mediated through a Bcl-2 family-triggered mitochondria-activated pathway (Fig 4). These results are similar with other topoisomerase 1 inhibitors such as camptothecin and irinotecan, which are also capable of modulating Bcl-2 family member expression and activating caspase-9 and -3, provoking mitochondrial apoptotic cell death in human cancer cells [65–67].

p53 has been well-recognized as a tumor suppressor by the finding that approximately half of all human tumors carry mutant p53 (loss of function or increased of oncogenic function) and served as a critical target in induction of cancer cell death [29]. Literature shows that p53 is one of the most important regulators in mediating growth arrest and apoptosis induced by various intrinsic or extrinsic stresses, including chemotherapeutic compounds [30]. The active p53 can transcriptionally increase the expression levels of p21^waf^ and p27^kip^ [39]. These proteins in turn stop the cell cycle progression by blocking the functionality of the cyclin-Cdk complex for cells to repair damages induced by various stresses. Once the damages are unable to be repaired, p53 activates the transcription of various pro-apoptotic genes, including Bax, Noxa, and PUMA [31] to execute the apoptotic process. Alternatively, p53 triggers apoptosis by repression of anti-apoptotic genes, such as Bcl-2, thus inducing the release of cytochrome c followed by the caspase activation [31]. Our results showed that austrobailignan-1-induced up-regulation of p53, p21^Cip1/Kip1^ and p27^Kip1^ in A549 cells was accompanied by G2/M arrest. However, austrobailignan-1-induced p21^Cip1/Kip1^ and p27^Kip1^ up-regulation as well as G2/M arrest were also observed in p53-knockdown A549 cells and a p53-null H1299 cells. Besides, increase of anti-apoptotic protein Bax and PUMA, decrease of pro-apoptotic proteins Bcl-2 and Mcl-1, activation of caspase cascade, and induction of mitochondrial-dependent apoptotic pathway were detected in both p53-wild-type and p53-null cell lines. Moreover, our results show that H1299 is more sensitive to the treatment of austrobailignan-1 than that of A549, suggesting that p53 is not necessarily required for austrobailignan-1-induced cell cycle arrest and apoptosis and further indicating that some other factors might be more critical than p53 in austrobalignan-1-induced cell cycle arrest and cell death. Similarly, several topoisomerase inhibitors have been shown to be able to cause cell cycle arrest and cell death irrespective of P53 status in various types of human cancer cell [68–70]. Our finding is important because the loss of functional p53 is reported to be found in more than half of cancer patients [33], and the broad range of signaling modules affected by austrobailignan-1 potentiates its application in cancer treatment. Several reports have mentioned that lignans induce cancer cell death accompanied with the activation of p53 [71–73]. However, honokiol induces the colorectal cancer cells death irrespective of p53 status [74]. These results demonstrate that different lignans might provoke a p53-dependent or -independent pathway in different types of cancer cell.

**Fig 7:**
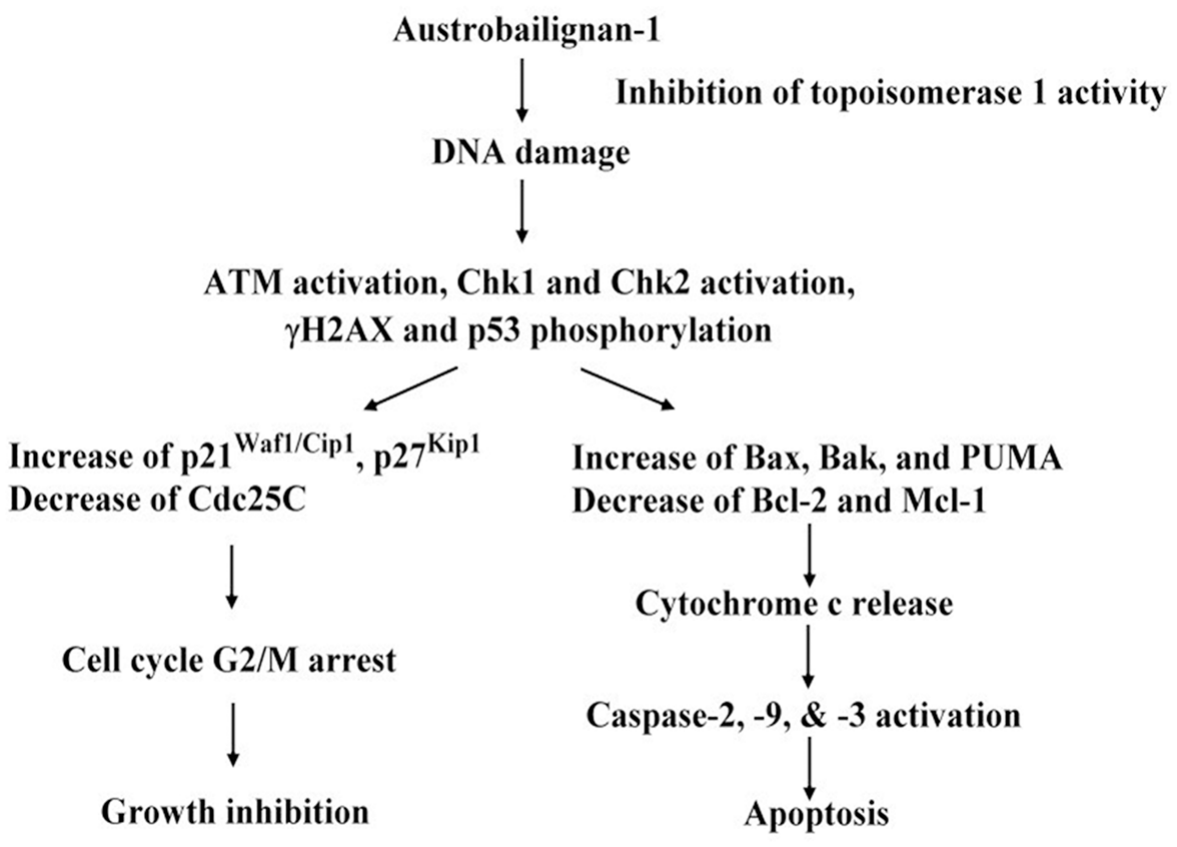
Schematic representation of the anti-cancer mechanisms of austrobailignan-1 in human non-small cell lung cancer A549 and H1299 cell lines.

Collectively, our observations provide evidence that austrobailignan-1, a lignan isolated from *Koelreuteria henryi*, was more potent than camptothecan in suppressing the topoisomerase 1 activity and inhibiting cell proliferation of human non-small cell lung cancer A549 and H1299 cells. Treatment of cells with austrobailignan-1 provoked a DNA damage response and induced the cell cycle arrest and apoptosis. Molecular and cellular mechanism studies revealed that austrabailignan-1 retarded cell cycle progression at G2/M phase through the ATM/Chks-Cdc25C, p21^Cip1/Kip1^ and p27^Kip1^ signaling pathways (Fig 7). Moreover, austrabailignan-1-induced apoptosis was via a Bcl-2 family protein-mediated mitochondrial death pathway (Fig 7). Besides, the relative lower working concentration of austrobailignan-1 compared with other traditional chemotherapeutic agents, such as cisplatin and doxorubicin (IC_50_ for A549 cells, cisplatin: 2–25 μM; doxorubicin: 2–5 μM, [75, 76]), makes it a potential chemotherapeutic candidate for the further study in the *in vitro* and *in vivo* models to determine the therapeutic efficacy and evaluate the potential of this compound for clinical applications.
